# Baseline results from the Norwegian radiology-led lung cancer screening pilot

**DOI:** 10.2340/1651-226X.2026.44910

**Published:** 2026-02-03

**Authors:** Albin Mahovkic, Kirill Neumann, Trond-Eirik Strand, Oluf Dimitri Røe, Haseem Ashraf

**Affiliations:** a Department for Diagnostic Imaging and Intervention, Akershus University Hospital, Lørenskog, Norway; b University of Oslo, Oslo, Norway; c Pulmonary Department, Akershus University Hospital, Lørenskog, Norway; d Department of Community Medicine, UiT – The Arctic University of Norway, Tromsø, Norway; e Department for Clinical and Molecular Medicine, Norwegian University of Science and Technology (NTNU), Trondheim, Norway; f Cancer Clinic, Levanger Hospital, Levanger, Norway

**Keywords:** Lung cancer, low-dose computed tomography, screening program, early-stage

## Abstract

**Background and purpose:**

Implementation of low-dose computed tomography (LDCT) screening for lung cancer is recommended. The Norwegian Lung Cancer Screening pilot (TIDL) has been conducted to explore recruitment strategy, detection rate and the value of a radiology-led screening program. This publication presents baseline results from the screened participants.

**Patient/material and methods:**

All 125,095 individuals aged 60–79 years in Akershus county, Norway were invited to participate. Ever-smokers completed a risk questionnaire based on the PLCOm2012_NoRace_ model: those with ≥35 pack-years or a ≥2.6% 6-year lung cancer risk were eligible for inclusion. Of 2,499 eligible participants, 1,006 underwent baseline LDCT between August 2022 and May 2023, and up to two more rounds later. Nodules were categorized by Lung-RADS v2022. Follow-up and staging were managed by thoracic radiologists; High-suspicion cases were referred to pulmonologists.

**Results:**

At baseline, lung cancer was diagnosed in 23 participants (2.3%), whereof 19 (83%) in stage I and 2 (9%) in stage II. Most underwent curative treatment, primarily robot-assisted surgery. Only 2.9% of the screened individuals were referred for further diagnostic evaluation. The false positive rate was 0.6% after pulmonologist referral and 1.7% after radiological staging. A total of 13.8% required 3- or 6-month imaging follow-up. Complication rates from diagnostic procedures were low.

**Interpretation:**

LDCT screening using combined risk-based eligibility and a radiology-led model is feasible and effective in the Norwegian context. The high detection rate and low clinical burden support its potential for national implementation. These findings may guide the development of future lung cancer screening programs in the Nordic countries and beyond.

## Introduction

Early detection of lung cancer through low-dose computed tomography (LDCT) has proven to significantly reduce morbidity and mortality by enabling diagnosis at an earlier and more treatable stage. This has been most convincingly demonstrated by two large randomized trials, the Dutch-Belgian NELSON study and the American National Lung Screening Trial (NLST) [1, 2]. Based on this evidence, the Council of the European Union has recommended initiation of pilot programs for lung cancer screening among individuals at high risk of developing lung cancer [3].

Identifying the appropriate target population is central to lung cancer screening and strongly influences both its effectiveness and cost-effectiveness [4]. Earlier studies defined eligibility using simple criteria such as age and cumulative smoking exposure (pack-years) [1, 2]. While such criteria are practical, they may fail to capture a substantial proportion of individuals who later develop lung cancer. Consequently, recent research and clinical practice increasingly emphasize risk prediction models that incorporate multiple factors, including smoking intensity, duration, quit-time, family history, comorbidities, and socioeconomic background [5, 6]. In Europe, the ongoing ‘4-IN THE LUNG RUN’ (4ITLR) study is exploring whether defining eligibility by specific high-risk profiles, rather than by age and smoking history alone, can improve cost-effectiveness and optimize outcomes [7, 8].

Another challenge relates to the organization of screening programs. Traditional screening models have been mostly pulmonologist-led, where pulmonologists are responsible for reviewing CT reports, initiating recalls, and managing follow-up examinations. While this ensures a high level of medical oversight, it is resource-intensive and may limit scalability [9, 10]. Could alternative organizational models, such as radiology-led follow-up, reduce the burden on clinical services while maintaining quality and safety standards?

Despite the strong evidence base and the increasing number of pilot programs across Europe, none of the Scandinavian countries have yet established a national lung cancer screening program. The Norwegian Lung Cancer Screening (TIDL) pilot study was therefore initiated in 2022 to assess the feasibility of LDCT-based screening in a Norwegian context. The study aims to assess not only detection rates and feasibility but also how novel eligibility criteria and a radiology-led protocol may contribute to a more resource-efficient implementation, using measures such as the false-positive rate, stage distribution and reduced referral burden on pulmonologists.

In this article, we present the results from baseline screening in TIDL of those selected for screening, with focus on management of CT findings, false positives, complications, and diagnosis, conducted in a population selected according to high-risk criteria. These findings may provide important insights into how a future national screening program could be adapted to national health care structures and patient populations.

## Patients/material and methods

### Study design and recruitment

This prospective pilot study was conducted at Akershus University Hospital (AHUS), which serves Akershus county with approximately 600,000 inhabitants in its catchment area. The hospital has the highest volume of lung cancer diagnostic work-up in Norway. Invitation letters were sent to all inhabitants aged 60–79 years. The invitation letter and information materials were reviewed by a user representative from a lung cancer patient organization, who provided feedback on how information could be communicated in a clear and balanced manner. Only ever-smokers, defined as having smoked at least 100 cigarettes or equivalent during their lifetime, were eligible for inclusion. Responders who returned informed consent completed a questionnaire based on the PLCOm2012_NoRace_ risk prediction model, which includes 10 factors: age, education, body mass index, history of chronic obstructive pulmonary disease, personal history of cancer, family history of cancer, smoking status, smoking intensity (cigarettes per day), smoking duration, and years since quitting for former smokers [5, 11].

### Study population

Eligibility criteria for participants in the study were the same as those used in the 4ITLR study for the possibility of future pooling of data. Participants were eligible for inclusion if they were current smokers or former smokers who had quit within the past 10 years and had either ≥35 pack-years of smoking history or a ≥2.6% 6-year risk of lung cancer according to PLCOm2012_NoRace_. Additional inclusion criteria were willingness and ability to comply with study procedures. Exclusion criteria included ongoing diagnostic work-up for recent abnormal pulmonary findings, current or prior history of lung, renal, melanoma, or breast cancer or body weight >140 kg. Eligible participants were randomized to either a control group or an intervention group with stratification for age and sex to ensure balanced distribution. From the intervention group, 1,000 participants were randomly selected and invited for baseline LDCT. Non-attenders were replaced by the next eligible participant on the list until the target of 1,000 scans was reached. Participants underwent scanning in up to three rounds: One group received annual screening (three rounds); the other received biennial screening (two rounds).

### LDCT acquisition protocol

Baseline scans were performed between August 2022 and May 2023. Examinations were performed on a Philips iCT MDCT scanner (Philips Healthcare). Participants were scanned supine, feet-first, after full inspiration, with low dose parameters.

### Image assessment

Images were primarily assessed by a thoracic radiologist (HA) with >10 years of experience. In uncertain cases, consensus was reached with other senior thoracic radiologists. Screen-detected nodules were managed according to Lung-RADS v2022 [12], with classification based on size and morphology. At baseline, indeterminate nodules 6–15 mm in diameter (113–1,767 mm³, Lung-RADS 3 or 4A) were scheduled for repeat LDCT at 3–6 months. Nodules >15 mm (>1,767 mm³, Lung-RADS 4B) were referred for diagnostic work-up.

Participants with indeterminate nodules (Lung-RADS 3–4A) remained in a radiology-led pathway, with follow-up LDCT scheduled by the radiologist. Growth was assessed using volume doubling time (VDT), measured semi-automatic in Philips Vue PACS with lung window. Nodules with VDT <600 days were considered high risk for malignancy [13]. This approach, integrating volumetric growth assessment, is consistent with other screening trials, including the Danish and NELSON studies [14, 15]. If findings progressed, participants were upgraded to Lung-RADS 4B and referred for further radiologic staging (PET-CT and/or full-dose contrast-enhanced CT of chest and upper abdomen). Participants with persistent Lung-RADS 4B category after staging were referred to the pulmonary department for clinical evaluation. Confirmed malignancies were discussed at multidisciplinary tumor boards to determine treatment strategy. Extrapulmonary CT findings were referred for further diagnostic follow-up by respective departments when indicated.

## Results

### Baseline characteristics

**Figure 1:**
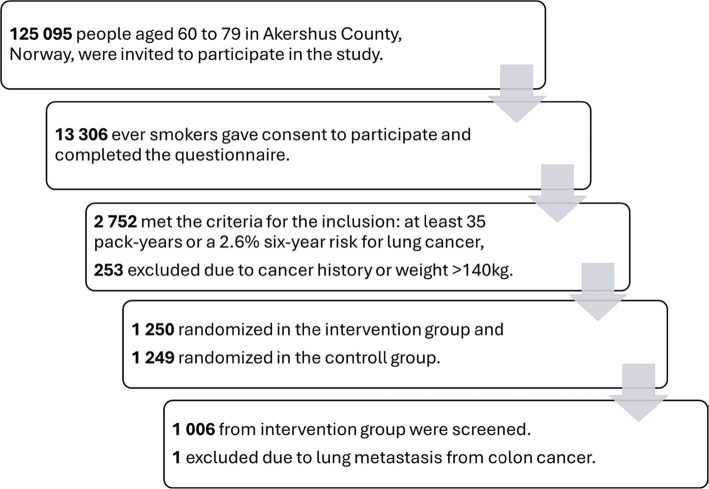
Norwegian Lung Cancer Screening Trial recruitment flowchart.

Of the 125,095 invited individuals, 13,306 (10.6%) consented to participate and completed a PLCOm2012_NoRace_ questionnaire. After eligibility screening and exclusion, 2,499 participants were randomized to intervention (*n* = 1,250) or control (*n* = 1,249) group. Of those in the intervention arm, 244 did not show up and thus the remaining 1,006 underwent baseline LDCT, whereof one participant was subsequently excluded due to prior malignancy (Figure 1). The mean age was 69 years, and 37.4% were female, detailed demographics are shown in Table 1.

**Table 1:** Demographics of participants undergoing LDCT in the Norwegian Lung Cancer Screening Trial (TIDL).

Age, average (SD)	69.1 (5.2)
Sex (%)	Males 629 (62.6)
	Females 376 (37.4)
Smoking intensity, average cigarettes per day (min–max)	19.2 (6–70)
Smoking duration (average years) (min–max)	44.6 (2–65)
Packyears (average) (min–max)	41.8 (4.2–135.0)
PLCOm2012_NoRace_ (mean) (min–max)	4.8 (0.5–46.2)

### Nodule follow-up

**Figure 2:**
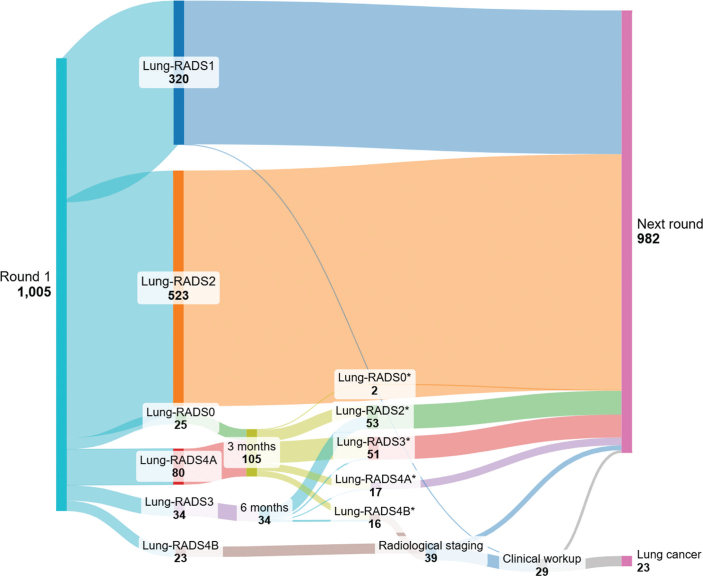
Nodule characterization and follow-up after baseline screening round (overview of classification according to Lung-RADS v2022 after baseline scan and after follow-up regimen).

At baseline, 83.9% of participants were classified as Lung-RADS 1–2, requiring no further work-up and subsequently were referred to the next screening round. Thirty-four participants were categorized as Lung-RADS 3 and scheduled for 6-month follow-up, while 105 were scheduled for 3-month follow-up (80 with Lung-RADS 4A and 25 with Lung-RADS 0). Twenty-three participants (2.3%) were classified as Lung-RADS 4B and referred directly for radiological staging (Table 2, Figure 2). At 3- and 6-month follow-up, a proportion of participants were upgraded to Lung-RADS 4B and referred for further diagnostic work-up. The majority of participants in these groups were downgraded to Lung-RADS 2 or 3, reflecting benign findings or resolution of infection-related changes.

**Table 2:** Lung-RADS after baseline scan and follow-up scans.

Lung-RADS	Baseline scan *n*	Follow-up regimen after baseline scan	Radiological staging *n*	3 months follow-up *n*	6 months follow-up *n*	Cancer diagnosed baseline*
0	25	3 months		2		
1	320	12 months				1
2	523	12 months	11	26	27	
3	34	6 months		48	3	1
4A	80	3 months		16	1	9
4B	23	Radiological staging		13	3	12
**Total**	**1005**			**105**	**34**	**23**

* Numbers in this column refers to initial category after first scan.

### Procedures and complications

A total of 31 CT-guided biopsies were performed on 25 participants, resulting in 19 malignancies (76%). Pneumothorax occurred in eight cases (25.8%), with one requiring chest drainage. In addition, 10 bronchoscopy procedures were performed on 10 participants and three lung cancers were diagnosed based on bronchoscopy alone. One cancer was diagnosed based on growth and PET-CT findings without biopsy due to poor lung function and high risk of complications.

### Lung cancer

In total, 23 participants (2.3%) were diagnosed with lung cancer during the baseline screening round. Histology included adenocarcinoma (73.9%), squamous cell carcinoma (17.4%), and large-cell neuroendocrine carcinoma (4.3%); one case (4.3%) was diagnosed radiologically without histology. Most cancers were detected at an early stage: 83% in stage I and 9% in stage II, while remaining 8% were in stage III (Table 3). After surgery 55% remained in same stage, while 35% were upstaged and 10% downstaged. Only one of 20 patients (5%) who underwent surgery had pathological stage III (Table 3). One cancer case was overlooked at baseline. The participant was diagnosed with locally advanced lung cancer 11 months after the baseline scan; this case was reported as a serious adverse event.

**Table 3:** Staging at diagnosis (TNM 8^th^ edition).

Clinical stadium	*n*	%	Pathological stadium*	*n*	%
T1aN0M0/stadium IA1	7	30.4	pT1aN0Mx/stadium IA1	5	25.0
T1bN0M0/stadium IA2	8	34.8	pT1bN0Mx/stadium IA2	8	40.0
T1cN0M0/stadium IA3	2	8.7	pT2aN0Mx/stadium IB	2	10.0
T2aN0M0/stadium IB	2	8.7	pT2bN0Mx/stadium IIA	1	5.0
T2bN0M0/stadium IIA	1	4.3	pT1aN1Mx/stadium IIB	1	5.0
T3N0M0/stadium IIB	1	4.3	pT3N0Mx/stadium IIB	2	10.0
T4N2M0/stadium IIIB	2	8.7	pT1bN2Mx/stadium IIIA	1	5.0
**Total**	**23**	**100.0**		**20**	**100.0**

* Pathological stadium for patients who underwent surgery.

Of those 23 participants diagnosed with lung cancer, four (17.1%) were included in the study based solely on a high 6-year risk of 2.6% or more and four (17.1%) were included based solely on a smoking history of 35 pack-years or more. The median 6-years risk for participants with a cancer diagnosis was 4.1%, compared to 3.8% for all screened participants.

### False positive rates

Of the 1,005 participants screened, 28 (2.8%) were referred for pulmonologist evaluation for clinical consultation and work-up after suspicious findings; 22 of these (78.6%) had lung cancer, corresponding to a false positive rate of 0.6%. In total, 39 participants (3.9%) underwent radiological staging, of whom 22 (56.4%) had lung cancer, corresponding to a false positive rate of 1.7%. If all indeterminate and suspicious nodules are considered positive, the overall false positive rate was 11.4%. In total, 139 participants (13.8%) were referred for 3- or 6-month follow-up.

### Treatment

Among participants with confirmed malignancy, 20 (87%) underwent robot-assisted thoracic surgery (RATS), one (4%) received stereotactic radiation due to poor lung function, and two received chemo-immunotherapy for locally advanced disease, whereof both passed away during the follow-up period. No other serious events were reported.

## Discussion and conclusion

This descriptive study reporting on the screening arm in the TIDL pilot demonstrated a relatively high detection rate of lung cancer at baseline LDCT screening (2.3%) in a selected group with particularly high estimated risk. The majority of cases were detected at an early stage (92%), and all the participants had a potentially curable stage. Almost all patients with early-stage disease underwent surgical resection, predominantly with RATS. The screening process was managed under a radiology-led workflow. The number of participants referred to clinical departments was minimal with only 2.9% of screened individuals undergoing pulmonologist evaluation. Using a clinically relevant definition of false positives – restricted to those findings referred to clinical work-up – the false positive rate was only 0.6%.

The detection rate in our trial was higher than reported in other trials. NLST reported 1.0% lung cancer detection at baseline, NELSON 0.9%, and DLCST 0.8% [16–18]. The most plausible explanation for the difference is the use of risk-based eligibility in TIDL, combining high age, high number of pack-years with the particularly high-risk cutoff of assessed with the PLCOm2012_NoRace_ model while other studies used just smoking history and lower age. Similar findings were observed in the Ontario Lung Cancer Screening Pilot, which used PLCOm2012-based selection with a threshold of 2.0% compared to 2.6% in the TIDL and reported a detection rate of 2.2% [9]. The estimated median 6-year risk of lung cancer in the TIDL intervention group was 3.8%, compared to 4.3% in the Ontario Pilot. The median risk for those with cancer diagnosis was 4.1% in the TIDL versus 8.7% in the Pilot. However, a higher risk threshold inevitably narrows eligibility and may exclude detection of several lung cancer cases. A recent Norwegian study estimated that the NELSON criteria, which are less restrictive than those used in TIDL, would capture only 37% of incident lung cancer cases over 6 years thus diminishing the effect the 24% survival gain found in NELSON in a real-world setting [6]. The use of a mixed model, as in our study, can detect more lung cancer cases, however its use has not been well studied. In TIDL, 17.1% of participants diagnosed with lung cancer would not have been included in the study if only one of the criteria has been applied. Thus, the choice of eligibility criteria is not a simple task, and this is probably the reason why there is still no international consensus on this matter.

The definition of a ‘positive finding’ strongly influences false positive rates. Some studies consider all Lung-RADS categories ≥3 as positive, which inflates the false positive rate given that most small nodules are benign [19]. We believe a more clinically relevant definition, counting only nodules that proceeded to diagnostic work-up is more relevant. By this definition, the false positive rate was 1.7% after radiological staging and 0.6% after clinical referral. This distinction matters because it better reflects the burden on patients and the health system. We argue that clinically focused definitions should be prioritized when assessing screening feasibility. Nevertheless, longer-term follow-up and results from subsequent screening rounds will be necessary to provide more robust estimates of false positive rates and to fully capture the cumulative impact of screening over time.

In TIDL baseline round, 83% of cancers were detected at early stage, higher proportions than reported in NELSON (64% stage I), NLST (53% stage I in the LDCT arm), and DLCST (53% stage I) [10, 17, 18, 20]. We attribute this not only to risk-based selection but also to the radiologist-led follow-up strategy, which facilitated timely biopsy decisions based on both nodule size and morphology. Complications related to CT-guided biopsies were low at 25.8%, which is even lower than in a larger study from our institution that reported 39% complication rate in non-screening context [21]. In our trial, a relatively high diagnostic yield suggests that experienced thoracic radiologists can safely shorten follow-up intervals and opt for early tissue diagnosis, thus reducing delays to treatment. These findings align with observations from the DLCST, where nodule morphology was emphasized as an important factor in clinical decision-making [10].

**Figure 3:**
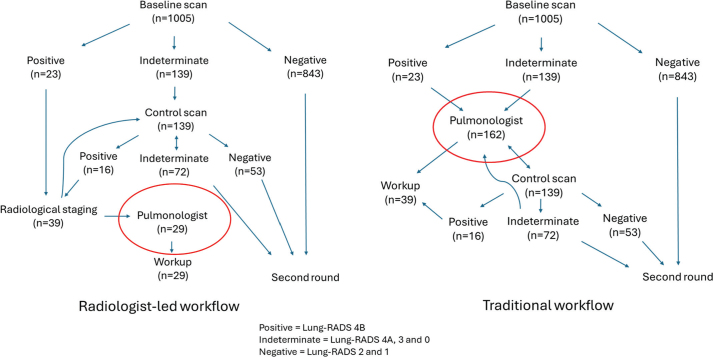
Radiologist-led versus traditional screening workflow.

A novel feature of TIDL was the radiology-led pathway, in which thoracic radiologists managed follow-up imaging, ordered diagnostic staging (PET-CT or contrast-enhanced CT), and referred participants to clinicians only when malignancy was strongly suspected. This organizational model has potential to reduce the workload for pulmonologists and other clinical departments, minimize delays, and allow for centralized expertise. Although this study was not designed to compare radiology-led management versus traditional management methods, based on the current results and experience it may be suggested this method is favorable. Only 29 participants (2.9% of those screened) entered the clinical pathway, of whom 23 (79%) had confirmed lung cancer. Compared to traditional screening workflow this approach could reduce pulmonologist referrals as shown in Figure 3. A radiology-led model may improve both efficiency and cost-effectiveness in large-scale programs. If implemented nationally, a radiologist-led approach could be organized with centralized assessment and triage by a pool of radiologists working in team, even remotely. The radiology-led model requires sufficient radiological capacity and expertise, which may not be universally available. Moreover, the radiology-led model as conducted in TIDL has been discussed within a multidisciplinary working and reference groups under the Directorate of Health, involved in planning a potential national lung cancer screening program and suggested as the preferred model [22].

The strengths of this study include its prospective design which reduces bias and enhances internal validity, and its use of mixed eligibility criteria combining smoking history with a validated risk prediction model. However, the study also has limitations, including the relatively small number of screened participants and restriction to a single county, which may limit generalizability. In addition, the combined use of clinical criteria and a risk model, as well as the selected PLCOm2012_NoRace_ threshold of 2.6 is not widely applied, which should be considered when comparing results across studies.

Future research should address issues central to national decision-making on lung cancer screening. This includes evaluation of the role of AI in CT analysis, as well as comparative studies of screening organization, with particular focus on resource use and cost-effectiveness. In parallel, several European countries are currently conducting pilot studies of lung cancer screening [23–25], highlighting the importance of generating robust evidence to inform policy decisions on the design and sustainable implementation of national screening programs.

## Conclusions

The TIDL pilot study demonstrated that LDCT screening with hybrid eligibility criteria in a high-risk Norwegian population yields a high baseline detection rate, with most cancers diagnosed at an early and potentially curable stage. The reported screening outcomes, including false positive rates and referral patterns, are described within a framework that applied a radiology-led diagnostic pathway. These findings supported by further research can contribute to developing the design of a future national screening program.

## Data Availability

The data set used in this study is not publicly available.
